# A New Complementary Touch for the Temporal Inverted Internal Limiting Membrane Flap Technique

**DOI:** 10.18502/jovr.v20.14516

**Published:** 2025-05-05

**Authors:** Levent Karabas, Ecem Önder Tokuç, Sevim Ayça Seyyar, Özlem Şahin

**Affiliations:** ^1^Department of Ophthalmology, School of Medicine, Kocaeli University, Kocaeli, Turkey; ^2^Department of Ophthalmology, School of Medicine, Gaziantep University, Gaziantep, Turkey; ^3^Department of Ophthalmology, School of Medicine, Marmara University, Istanbul, Turkey

**Keywords:** Inverted Internal Limiting Membrane Flap Technique, Macular Hole, Vitrectomy

## Abstract

The temporal inverted internal limiting membrane (ILM) flap technique was developed to improve vitreoretinal surgery for large macular holes (MH). However, in addition to the difficulty of the surgical procedure, the main concern is the displacement of the ILM flap due to small fluid leakage into the posterior pole, even in the short time required to close the sclerotomies after fluid–air exchange. A new approach to the temporal inverted ILM flap technique is described here. In this approach, when the ILM flap is inverted over the MH, ILM forceps, while it is closed, are gently pressed over the folded edge and passed over (just like folding a paper in half), creating an ILM fold mark like the ones used in origami. Thus, it can be seen that the minimal fluid leaking into the posterior pole ventilates the free edge of the flap, but the force formed along the folded edge prevents the flap turnover.

##  INTRODUCTION

Through the pioneering study of Kelly and Wendel, published in 1991, it was discovered that full-thickness macular holes (MH), previously thought to be incurable, can be treated with pars plana vitrectomy (PPV) and liquid gas exchange.^[1]^

The addition of internal limiting membrane (ILM) peeling to MH surgery and the introduction of various staining agents for better viewing of the ILM have significantly increased success rates over the years.^[2,3]^ Although all these advances are revolutionary in the treatment of MH, there is still a relatively high risk of surgical failure for large ( > 400 μ m) holes.^[4]^ To overcome this problem, Michalewska et al^[5]^ developed the inverted ILM flap technique for primary surgical repair of large MH in 2010. In addition to the significant differences in skill level from surgeon to surgeon in ILM flap techniques, another issue that needs to be addressed is the stabilization of the created ILM flaps on the fovea. In the short time interval when sclerotomies are closed after the air–fluid exchange is performed with maximum care, even minimal fluid collected in the posterior pole may cause flap displacement. We describe an additional step that we believe is important for achieving maximum flap stabilization in MH surgery performed with the temporal inverted ILM flap technique.

##  SURGICAL METHOD

The technique was performed in patients with macular holes larger than 400 μ m, those with idiopathic primary MHs and no history of prior ILM peeling.As an additional step to the temporal inverted ILM flap technique, gently pressing over the flap, like massaging, along the folded edge of the flap with closed-ended ILM forceps or the tip of the vitrectomy probe has been described to maintain flap stabilization. The surgical procedure included a wide-angle non-contact imaging system (Resight 700; Carl Zeiss Meditec AG, Jena, Germany) for the modified ILM flap technique and a 23-G PPV (OS4; Oertli Instrumente AG, Berneck, Switzerland) using 16% Sulphurhexafluoride (SF6; sulphurhexafluoride, Teknomek Medical Products, Istanbul, Turkey) gas tamponade. At the beginning of the operation, a double chandelier was placed to control the stabilization of the ILM flap at the end of the surgery. After core vitrectomy, the posterior vitreous detachment was created by aspiration with a vitreous cutter in the eyes where the posterior hyaloid is not detached, and a better visualized posterior hyaloid was cleared entirely by using triamcinolone when necessary. First, if the patient has an epiretinal membrane, it is stained and peeled off. Next, 0.025% Brilliant Blue G-stained ILM (view ILM, Alcimihia, Italy) was peeled off, starting from the temporal side, approximately two optic discs from the center of the fovea, toward the superior and inferior vascular arcuates, to form free edges. The ILM, which was held by the free edges, was carefully peeled toward the MH to form a flap almost adjacent to the hole. The created ILM flap was inverted over the MH using an injection of perfluorocarbon (PFCL; Perfluorodecalin, Teknomek Medical Products, Istanbul, Turkey). PFCL, with its high density and interfacial tension, stabilizes the inverted free ILM flap on the MH and makes the folded edge clear (similar to folding a sheet of paper in half). Then, it was passed over the folded edge several times by gently pressing it with closed-ended ILM forceps or the tip of the vitrectomy probe. After gently massaging the crease line of the ILM flap, a fluid–air exchange was performed first, followed by a careful PFCL–air exchange. Finally, the air–SF6 exchange was performed, and the sclerotomies were closed. Before removing the double chandelier, the amount of fluid accumulated in the macula and the position of the ILM flap were checked [Video 1]. Then, the patients are quickly brought to the prone position from the operating table and transferred to the transfer stretcher. The patient was recommended to remain in the prone position for 3–7 days.

**Video 1.**

The Brilliant Blue G-stained ILM is peeled from the temporal side to the superior and inferior vascular arcuates to form free edges. The ILM flap is inverted over the macular hole with the help of perfluorocarbon injection. Then, it is passed over the folded edge by gently pressing with closed-ended ILM forceps. PFCL–air exchange and the air–SF6 exchange are performed and the sclerotomies are closed. In the final stage, the double chandelier image shows no displacement of the ILM flap.

##  RESULTS

**Figure 1:**
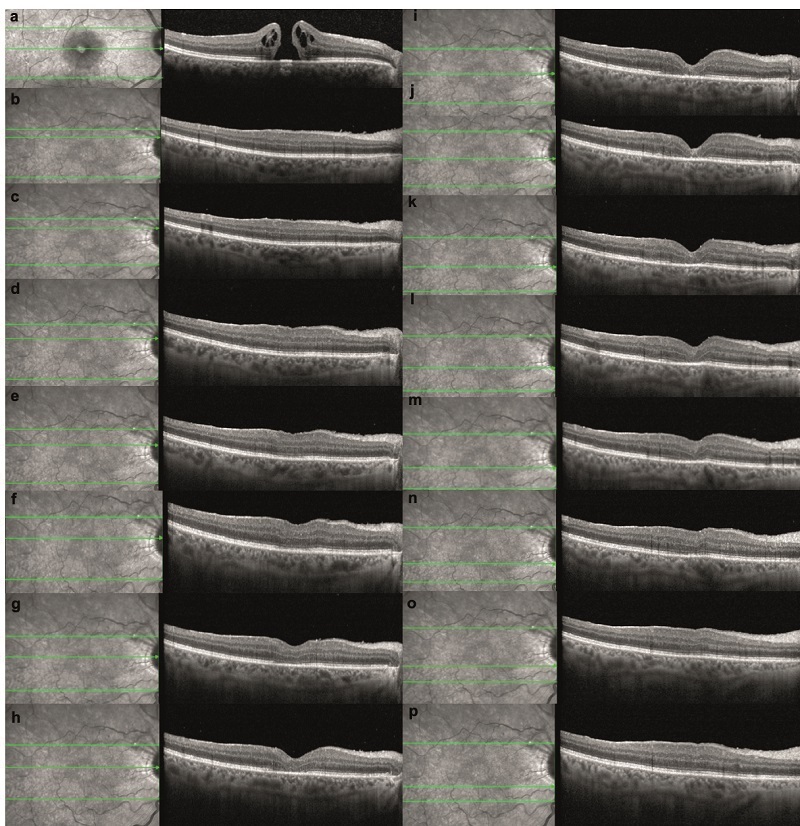
Macular hole in the right eye of a 53-year-old woman. (a) The preoperative OCT image shows a macular hole with elevated edges and intraretinal cystic spaces. (b–p) Consecutive early postoperative OCT scans indicate successful closure of the macular hole, with no evidence of photoreceptor or inner retinal damage.

**Figure 2:**
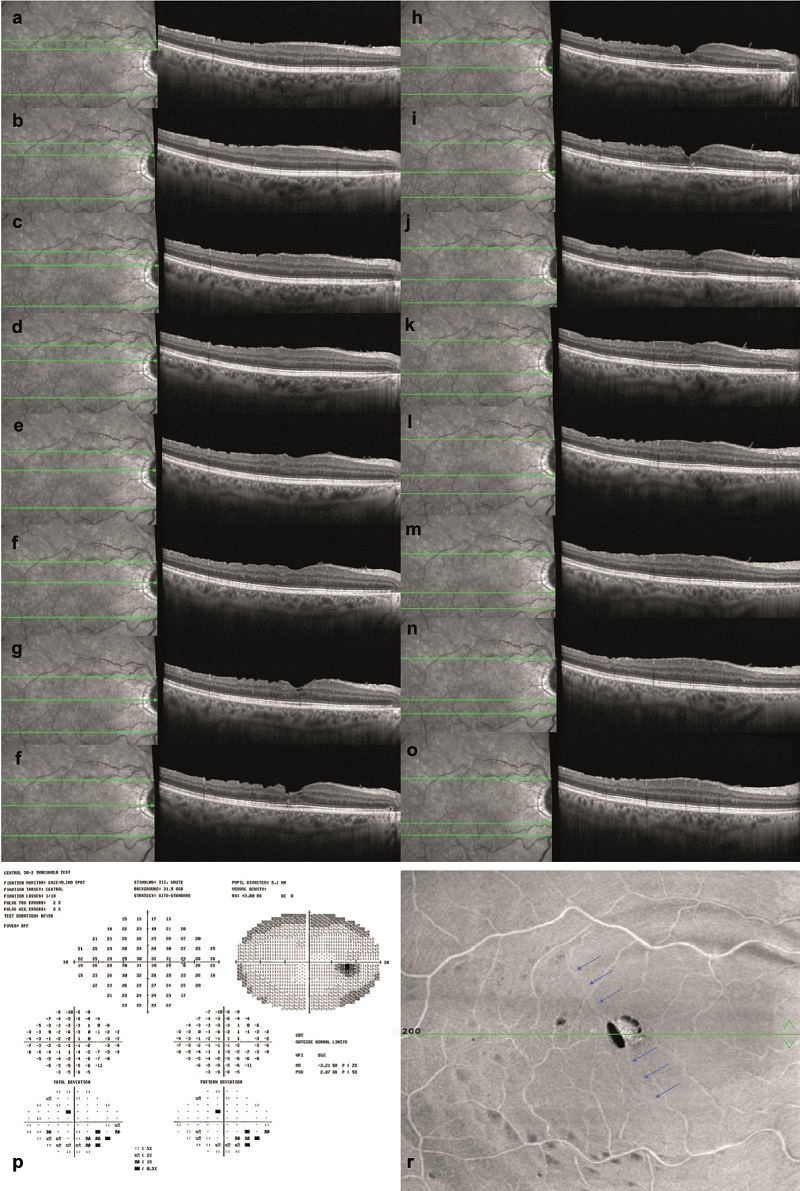
(a–o) Sequential postoperative late-period OCT scans demonstrating significant improvement in the ELM and EZ. It is evident that the integrity of the EZ and ELM is maintained, with no observable damage in the inner and outer retinal layers following the ILM flap massage. (i) The central visual field of the right eye shows a low degree of sensitivity loss and a frame defect below the blind spot. (p) There is no loss of sensitivity around the fovea and in the ILM fold line. (r) Blue arrows denote the fold line of the ILM flap in the en-face image.

Patients were operated between January 2021 and August 2022. Fifty-five eyes of 54 patients were operated on with the assisted technique described above. The characteristic features of the patients are shown in Table 1. When checked with the chandelier light at the end of the surgery, we observed fluid collection filling between the inferior and superior arcuates in all patients. The ILM flap was not displaced in any of the patients until the closure of the sclerotomies. When the gas was resolved, Optical Coherence Tomography (OCT) and en-face images revealed no flap displacement in patients [Figures 1 & 2].

**Table 1:** Characteristics of the patients

Patients (eyes), *n*	54, (55)
Age, yrs, mean ± SD	64.8 ± 5.2
Gender, *n* (%)	
Female	23 (42.5)
Male	31 (57.5)
BCVA, logMAR, mean ± SD	
Preoperative	0.67 ± 0.20
Last follow-up	0.29 ± 0.32
Diameter of MH, µm, mean ± SD	642.65 ± 94.85
Preoperative ERM, (eyes), *n*	7, (7)
The time interval between the air-SF6 exchange and the closure of sclerotomies, mins, mean ± SD	2.53 ± 1.20
Subsequent Sx	–
Complication	–
Hole closure, (eyes), *n*	54, (55)
Follow-up duration, months	10.34 ± 6.5
BCVA, best-corrected visual acuity; ERM, erpiretinal membrane; Sx, surgery; MH, macular hole; SF6, sulphur hexafluoride; Mins, minute	

##  DISCUSSION

This article describes a novel modification of the temporal inverted ILM flap technique, consisting of massaging the folded edge of the ILM flap to improve flap stabilization. In our practice, in all cases where we used the temporal inverted ILM flap technique, we brought the free flap closer to the fovea by gently pressing and pinching the folded edge formed when the ILM flap was folded over the fovea. Using this method, we created a folding sign in the folded ILM flap, similar to folding a piece of paper in half to stabilize the flap. Our clinical experience using this technique has yielded excellent results regarding postoperative ILM flap stabilization.

Acting as a scaffold, almost like a basement membrane, for the proliferation and migration of müller cells, ILM flaps have been advocated to facilitate hole closure by allowing photoreceptors to assume the proper position during the reconstruction process. Several variations of the reverse ILM flap technique exist, including differences in flap size, shape, number, and how flaps are closed over MHs.^[5,6,7,8,9]^ In addition, various aids such as perfluoro-n-octane, viscoelastics, and macular plug formation with autologous blood are used to stabilize the free or inverted ILM flap in the appropriate place until the end of the fluid–air exchange.^[10]^ All these modifications aim to overcome concerns about the inverted ILM flap technique. These concerns include the surgical procedure being quite tricky and the steep learning curve, as the ILM has to be removed, leaving the remnants attached to the edge of the MH. A final concern is that the ILM flap may be displaced, especially during the fluid–air exchange. Even after gas induction, even minimal fluid that collects in the posterior pole during the time it takes to close the sclerotomies may cause the flap to dislocate before the patient is in the appropriate end position. Spontaneous retroversion of the ILM flap during the fluid–air exchange was reported in 14% of cases in the original reverse ILM flap technique.^[5]^ Even if surgical failure is due to multiple factors, the original technique needs to be further improved by anticipating any risk that may impair flap stabilization and developing strategies against all risks.

In addition to various previously reported modifications of ILM flap techniques, we observed that fluid accumulated in the fovea during the closure of sclerotomies after air–fluid exchange and gas induction could cause the flap to be dislodged. Thus, we sought a modification to avoid this complication. We imagined the ILM flap as a piece of paper that we would fold in half. In the original technique, the macular hole was massaged 360º from all sides, and then the ILM was compressed into the hole.^[5]^ However, by massaging the flap along the folded edge, it would be compressed toward the fovea, and a force would be created that prevents it from floating freely by detaching it from its place. In cases where this technique was applied, the flap position will still be as desired, even if the minimal fluid leaking into the posterior pole during sclerotomy closure slightly ventilates the free edge of the flap. As a result of our clinical experience, when none of the 54 patients treated with this technique experienced ILM displacement, we suggest that this modification will benefit patients undergoing this surgery.

Displacement of the flap during or after fluid–air exchange causes surgical failure, which must be resolved.^[10,11]^ Our clinical experience suggests introducing this modification when performing ILM surgery reduces the surgical failure rate. We recommend that more extensive studies confirm our encouraging findings.

##  Financial Support and Sponsorship

None.

##  Conflicts of Interest

None.
